# Distinctive Lipogenic Gene Expression Patterns in the Mammary Glands of Dairy Cows Are Associated with the Unique Fatty Acid Composition of Bovine Milk Fat

**DOI:** 10.3390/foods14030412

**Published:** 2025-01-27

**Authors:** Roni Tadmor-Levi, Nurit Argov-Argaman

**Affiliations:** Department of Animal Sciences, RH Smith Faculty of Agriculture, Food and Environment, The Hebrew University of Jerusalem, Rehovot 7610001, Israel

**Keywords:** dairy, *de novo* synthesis, triglycerides, lipids, AGPATs, Acyl-CoA synthase, adipose

## Abstract

Fat composition is largely responsible for the technological and rheological properties of cow milk and dairy products. Bovine milk fat is unique in terms of its fatty acid composition and positional distribution, with about 25% of its fatty acids being short- and medium-chain, which are synthesized *de novo* in the mammary gland and are not present in extra-mammary tissues. With the aim to identify potential genetic factors responsible for the unique composition of bovine milk fat, we extracted genes with GO annotations related to lipid metabolism and performed a gene expression mega-analysis. Overall, different lipogenic tissues (i.e., mammary, liver, and adipose) displayed discerned expression patterns. In a PCA, the liver was significantly separated from adipose and mammary tissues. In a correlation analysis with the fatty acid synthetase (FASN) gene, notable differences among the tissues were found. In the mammary gland, the majority of genes (~70%) were negatively correlated with FASN expression, whereas only 18% were negatively correlated in adipose. Only a few genes were positively correlated with FASN exclusively in the mammary gland, including AGPAT1 and AGPAT6, which also had the highest expression in the mammary gland compared with adipose. Looking at the expression levels in tissues (TPM) revealed significant differences in the expressions of genes responsible for the activation of fatty acids by ligation to CoA, according to their carbon chain length. Notably, the ACSS1 gene, which converts acetate to acetyl-CoA, had the highest expression in the mammary gland, whereas genes responsible for the activation of long-chain fatty acids had lower expressions. The findings of the present study suggest that the unique properties of dairy fat are the results of the distinct expression patterns of genes involved in *de novo* synthesis of fatty acids and their downstream utilization.

## 1. Introduction

Besides nursing infants, humans consume milk almost solely from ruminants. Milk fat consists of 98% triglycerides (TAGs) with a molecular structure of three fatty acids esterified to a glycerol backbone, and the remainder of the lipid mass consists of polar lipids and cholesterol [1]. Ruminant milk used for dairy products has a unique TAG composition that consists of a blend of saturated, mono, or polyunsaturated fatty acids, and long-, medium-, and short-chain fatty acids, with relatively high concentrations of the latter, which can make up over 20% of the total fatty acids [2]. Fatty acids are not randomly esterified to the three positions on the glycerol backbone. In dairy fat, short-chain fatty acids are prominently esterified to the sn-3 position, while medium- and long-chain fatty acids are more variable in their positions [3]. This composition is distinguished from that of non-ruminant milk and non-mammary ruminant lipogenic tissues, such as liver and adipose tissue. The unique chemical characteristics of ruminant milk fat are responsible for the unique technological (such as the solid fat content under physiological temperature) and rheological properties of dairy products, with a distinguished texture, mouthfeel, and taste [4]. In addition, the release of short-chain fatty acids through hydrolysis at the sn-3 position, catalyzed by lingual lipase, contributes to the experience induced by the consumption of these fats and not by oils or other animal fat sources (meat) [5].

In recent years, the demand for sustainable alternatives to animal-based foods has significantly risen. However, these alternatives often struggle to mimic the flavor, aroma, and mouthfeel of animal-based foods. While protein alternatives are more easily produced using precision fermentation [6], replacing lipids is more challenging since they are a blend of products of several biochemical pathways. Without a profound understanding of the mechanisms underlying these processes in the mammary gland of ruminants, the success rates are expected to remain limited.

The production of milk TAGs is a multistep process that can generally be divided into three parts. The first part involves providing lipogenic building blocks (mainly acetyl-CoA and malonyl-CoA) for fatty acid synthesis, the second part involves the *de novo* synthesis of fatty acid chains and chain termination, and the third part involves the utilization of fatty acid products by acyltransferases to produce TAGs. While long-chain fatty acids are partially provided to the mammary gland by the blood stream, the short- and medium-chain fatty acids in milk are entirely synthesized *de novo* [7]. The *de novo* synthesis of fatty acids is a highly conserved mechanism that is used by every lipogenic tissue and by a variety of ruminants and non-ruminant species [8]. This mechanism involves the loading of acetyl-CoA onto the fatty acid synthetase complex (FASN), followed by elongation steps, in which two carbons, acquired from malonyl-CoA, are added. Malonyl-CoA is produced by acetyl-CoA carboxylase (ACAC), which is considered the rate-limiting step in lipid synthesis [9,10]. The elongation of fatty acids by FASN is typically terminated when the chain length reaches 16 carbons (C16), and fatty acids are released by the thioesterase catalytic site of FASN.

Interestingly, the inhibition of thioesterase does not affect the synthesis of short-chain fatty acids by FASN isolated from bovine tissues; thus, short-chain fatty acid synthesis may be independent of thioesterase [11]. Hansen and Knudsen (1980) suggested that the bovine transacylase catalytic site on FASN has an extended affinity for C4:0, C6:0, and C8:0 beyond the common affinity for acetyl-CoA (C2). This property was hypothesized to enable the release of short-chain fatty acids by reversing the loading reaction. In bovines, there is only one active FASN transcript [12]; thus, the same unique transacylase unit of FASN is active in all tissues. However, there is a profound inter-tissue difference in the fatty acid composition, as the significant percentage of short- and medium-chain fatty acids (≤c14:0, making up approximately 25% of total fatty acids) is unique to the mammary glands and milk of ruminants and is not found in other tissues [2,13,14]. Therefore, the unique abundance of short- and medium-chain fatty acids in bovine milk is not attributed to differences in FASN alone, and they are hypothesized to be the product of a unique production apparatus in the mammary gland, which is distinguished from other tissues. In this study, we used a mega-analysis approach to analyze RNA-seq data from publicly available resources and characterize the distinctive transcriptional fingerprints that may contribute to the unique fatty acid profile produced by the mammary gland.

## 2. Materials and Methods

### 2.1. Data Acquisition

For the expression analysis, we analyzed a comprehensive list of genes from the lipogenic pathway. The genes in the dataset were selected to include genes related to the following GO terms: GO:0006629 (lipid metabolic process), GO:0034440 (lipid oxidation), GO:0016297 (fatty acyl-ACP hydrolase activity), GO:0047617 (fatty acyl-CoA hydrolase activity), GO:0005504 (fatty acid binding), GO:0042975 (peroxisome proliferator activated receptor binding), GO:0035357 (peroxisome proliferator activated receptor signaling pathway), GO:0015910 (long-chain fatty acid import into peroxisome). Additionally, we validated that all known genes from the following categories were included and not missed: fatty acid *de novo* synthesis genes (ACACA, ACACB, and FASN), triglyceride (TAG) biosynthesis genes [glycerol 3-phosphate acyltransferases (GPATs), 1-acylglycerol-3-phosphate-O-acyltransferase (AGPATs), and diacylglycerol O-acyltransferase (DGATs)], and acyl-CoA synthases short (ACSS) and long (ACSL) family members. This gene list included 779 genes. Based on the GO annotations and gene descriptions, we divided these genes into six super categories: (i) availability of building blocks for the *de novo* synthesis of fatty acids, (ii) acyl-CoA thioesterases, (iii) elongases and desaturases, (iv) acyl-CoA synthases, (v) phospholipid and sphingolipid biosynthesis, and (vi) TAG biosynthesis (Supplementary File S1).

Expression data for selected lipogenic genes were extracted in the form of raw count data from the Cattle Genotype-Tissue Expression Atlas [15,16] and converted to transcripts per million (TPM) normalized read counts [17]. Genes with a TPM lower than 1 across all selected samples were excluded from the analysis, resulting in a final list of 735 genes. The samples were filtered based on their tissue of origin (adipose, liver, mammary gland, and milk cells) and quality parameters (a read count per sample above 0.5 M and a mapping rate above 60%). Additionally, to reduce interspecific variation, samples of bovine species other than *Bos taurus* were excluded. Since only females produce milk, non-female samples were also excluded. Several samples of lactating mammary glands from BioProject PRJEB30263 contained many missing data for most genes; therefore, they were also removed from the final analysis. Altogether, the analysis focused on 65, 149, 136 and 156 adipose, liver, mammary, and milk cell samples, respectively. The information of all samples and analyzed genes is provided in Supplementary File S1.

### 2.2. Data Analysis

For analysis and presentation, all TPM data were log transformed [LOG(TPM+1)], and the means and standard errors were calculated for each gene and tissue (see Supplementary File S2 for the summary statistics of each gene and tissue). All statistical analyses were performed using JMP pro 17.0.0 software (SAS). Principal components analysis (PCA) was performed on standardized values, based on covariances. Significance values were corrected for multiple comparisons using the false discovery rate (FDR) method [18]. Differences in the mean gene expressions among tissues were determined using the Kruskal–Wallis test followed by all-pair comparisons using the Steel–Dwass method (see Supplementary File S3 for Steel–Dwass comparisons and their respective *p*-values). Hierarchical clustering of genes was performed based on the correlation coefficients (r) between FASN and selected lipogenic genes in adipose and mammary tissues (see Supplementary File S4 for all correlation coefficients and their respective *p*-values). Venn diagrams were illustrated using Venny 2.0.1 [19].

The final dataset, including all analyzed genes, samples, sample metadata, raw reads, TPM and Log(TPM+1) for each sample and gene, is provided in Supplementary File S1.

## 3. Results

### 3.1. Overview of Lipid Metabolism Genes in Analyzed Tissues

We performed a mega-analysis on the expression data of lipid metabolism-related genes in bovine mammary glands and milk cells and compared them to those in other lipogenic tissues (adipose and liver). The final dataset included 65, 149, 136, and 156 adipose, liver, mammary, and milk cell samples, respectively.

**Figure 1:**
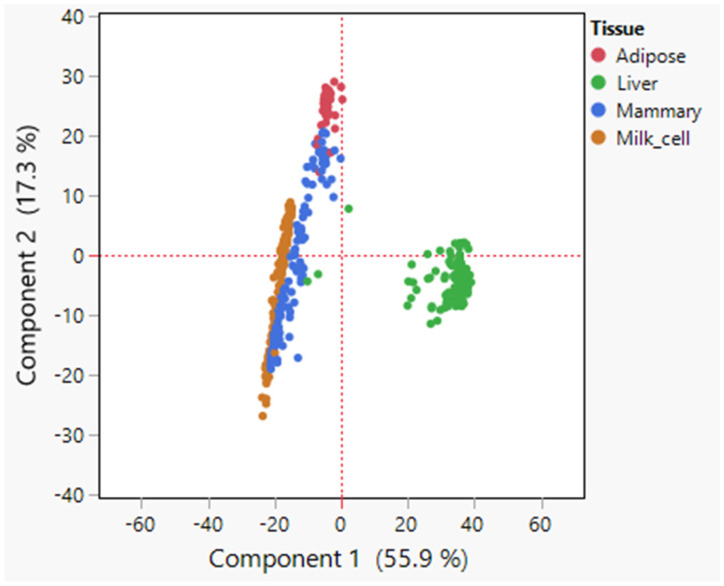
PCA analysis of expression data. Principal component analysis (PCA) plot, based on covariances, demonstrating differences in lipid metabolism gene expression across lipogenic tissues (adipose, mammary, liver, and milk cells). The PCA analysis revealed a distinct separation of the tissues in the reduced dimensional space defined by the principal components. Each data point represents a tissue sample, with different colors indicating different tissues.

Initially, principal component (PC) analysis was performed on the expression data of the 735 lipid metabolism genes across samples of the abovementioned tissues (Figure 1). The strongest separation observed was between the liver samples and all other tissues along the PC1 scale, accounting for 55.9% of the total variance in the data. Furthermore, there was some separation between adipose tissue and the mammary gland and milk cells samples along the PC2 scale, which explained 17.3% of the total variance (Figure 1). The mammary tissue samples demonstrated some separation along the PC2 axis, with some samples more similar to adipose tissue samples and other more similar to milk cells (Figure 1). These differences could be attributed to different stages of the lactation cycle and different sampling procedures, which were not provided with the data.

These results suggest that these tissues display discernible differences in their gene expression profiles related to lipid metabolism, which could explain the differences in their fatty acid compositions. Due to the significant separation of the liver from the other samples (Figure 1), and since almost no fatty acid synthesis occurs in bovine liver and other lipid metabolism processes (e.g., oxidation of lipids) are more prominent [20], we focused on adipose and the mammary gland for further analysis.

### 3.2. Correlation Patterns with FASN Gene Expression

FASN, as the enzyme responsible for the *de novo* synthesis of fatty acids, is expected to be expressed at higher levels in cells where the production of fatty acids is occurring. Furthermore, other genes encoding for enzymes responsible for upstream and downstream processes related to the production, modification, and utilization of fatty acids are expected to be co-expressed with FASN. However, as demonstrated above, different tissues display different expression patterns that may contribute to the different fatty acid compositions among them. To further study the expression patterns of genes within the same pathways, we examined the correlations between the expressions of these lipid metabolism genes and that of the FASN gene in adipose and mammary tissues.

**Figure 2:**
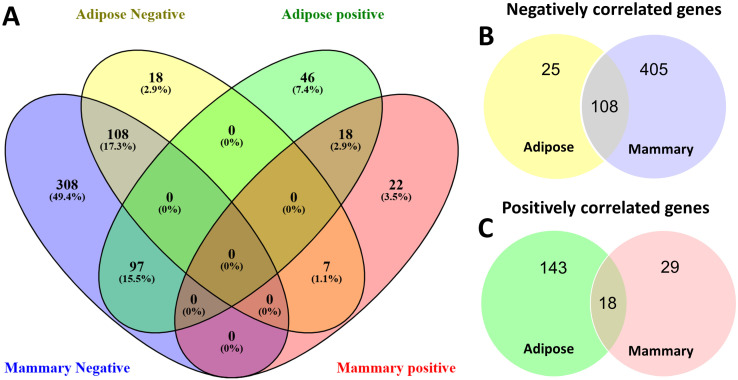
Venn diagram based on correlation with FASN gene expression. Gene expressions of 734 genes with GO annotations related to lipid metabolism were correlated with FASN gene expression in adipose and mammary tissues. A total of 622 genes that were significantly correlated with FASN in adipose and/or mammary are visualized in Venn diagrams. (**A**) Venn diagram of both positively and negatively correlated genes in adipose and mammary (illustrated using Venny2.0.1 [19]). (**B**,**C**) Venn diagrams of negatively (**B**) and positively (**C**) correlated genes in adipose and mammary.

Overall, the two tissues strongly differed in the way in which the expression of their lipid metabolism genes correlated with FASN expression (Figure 2, Supplementary File S4). The most notable difference was in the general direction of the genes’ correlation with FASN. In the mammary gland, 513 genes out of 734 (69.9%) lipid metabolism-related genes were negatively correlated with FASN (Figure 2B). Of these genes, 78.9% were unique to the mammary gland and either positively correlated (97 genes) or not significantly correlated (308 genes) in adipose (Figure 2A). Among these genes were AGPAT2, AGPAT4, ACSL4, and more (Supplementary File S4). In contrast, only 25 genes in adipose were uniquely negatively correlated with FASN expression (Figure 2B), and 7 of those (e.g., AGPAT1) displayed opposite patterns in the mammary gland, with positive correlations (Figure 2A, Supplementary File S4).

We focused on the genes displaying a positive correlation with FASN that were shared or unique for each tissue. Only 18 genes were positively correlated in both tissues (Figure 2C). Notably, among these genes were ACACA, ACSS2, ACSL1, GPAM, and SCD (Supplementary File S4). On top of those, 29 and 143 genes were positively correlated uniquely in the mammary gland and adipose tissue, respectively (Figure 2C). We examined all gene lists with a positive correlation with FASN from adipose and mammary tissues (Supplementary File S4), along with their full GO term annotations, and then selected molecular functions directly related to the production of different fatty acid profiles, which belong to the six categories described in the methods section (availability of building blocks for *de novo* fatty acid synthesis, acyl-CoA thioesterases, elongases and desaturases, acyl-CoA synthases, phospholipid and sphingolipid biosynthesis, and TAG biosynthesis). Notably, of these six categories, only two are represented in genes that are positively correlated with FASN in the mammary gland; however, all categories are represented in adipose (Table 1).

**Figure 3:**
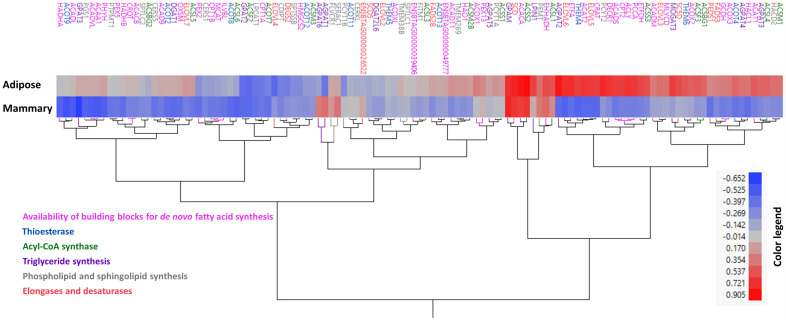
Clustering of candidate genes based on correlation with FASN gene expression. Hierarchal clustering analysis of 115 analyzed genes from six functional categories [availability of building blocks for the *de novo* synthesis of fatty acids (pink), acyl-CoA thioesterases (blue), elongases and desaturases (red), acyl-CoA synthases (green), phospholipid and sphingolipid biosynthesis (grey), and TAG biosynthesis (purple)] based on their expression level correlations coefficients with FASN gene expression level in adipose and mammary tissues. Genes are presented with their name, or if absent, with their stable IDs. Text color refers to the category according to the legend.

Next, we extracted a gene list of 115 genes from these six categories (as mentioned in Supplementary File S4). Clustering analysis of these genes based on their correlation patterns with FASN in adipose and mammary did not cluster genes together based on function or annotation (Figure 3). Genes from the different categories were separated into different clusters, and interestingly, different isoforms in gene families were not clustered together (for instance, AGPATs did not cluster together; Figure 3). The fact that different isoforms clustered separately indicates that they behave differently (both in expression and potentially in relation to fatty acid production) in different tissues. We assume that the distinct fatty acid composition in each tissue partly stems from these differences.

### 3.3. Analysis of Expression for Specific Genes

Next, we analyzed the expressions of genes from all six categories in mammary, adipose, and milk cells. The results of all genes are displayed in Supplementary File S2. Except for one desaturase gene (SCD5), which was significantly higher in the mammary gland (Steel–Dwass test, *p* < 0.05), in the three categories of “elongases and desaturases”, “availability of building blocks for *de novo* synthesis”, and “phospholipid and sphingolipid biosynthesis”, all genes were expressed at similar or higher levels in adipose tissue. The genes from these categories are not further discussed since their potential contribution to the production of short-chain fatty acids is not well described.

We further focus on the three categories that consist of genes with potential direct contributions to the length of *de novo* synthesized fatty acids: (i) thioesterases, (ii) acyl-CoA synthase genes, and (iii) TAG synthesis genes.

#### 3.3.1. Thioesterase Genes

**Figure 4:**
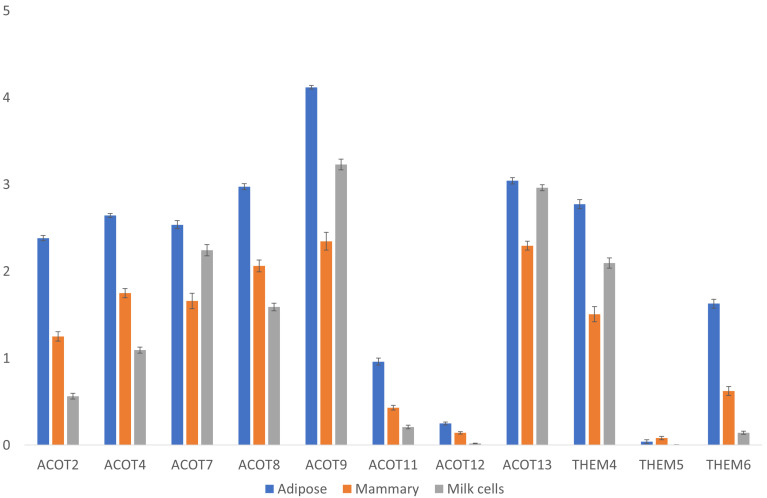
Expressions of thioesterase genes. Expressions [Log(TPM+1)] of acyl-CoA thioesterase genes in adipose and mammary tissues and milk cells. Means and error bars of all samples are shown by gene and tissue. Statistical analysis of the differences among the means of different tissues for each gene are provided in Supplementary File S3.

In addition to the thioesterase catalytic site of FASN, other separately encoded thioesterases might be involved in “releasing” the fatty acid carbon chain from the acyl carrier protein (ACP) unit of FASN at varying chain lengths. Therefore, acyl-CoA thioesterase gene expression was also analyzed (Figure 4). Its expression in the mammary gland was significantly lower than that in adipose tissue for all genes (Steel–Dwass test, *p* < 0.05), except for the THEM5 gene, which had a similar and very low expression in both tissues (Figure 4). Interestingly, its expression in milk cells was sometimes lower and sometimes higher than that in mammary glands and significantly lower than its expression levels in adipose for all genes (Steel–Dwass test, *p* < 0.05), except for ACOT13 and ACOT7, for which the expression levels were similar (Figure 4). Since these milk-derived cells contained various populations of different cell types, their mean expressions of different genes varied considerably compared with the tissue samples derived from the mammary gland. Overall, none of these thioesterases were unique to or highly expressed in the mammary gland or in milk cells compared with adipose tissue (Figure 4). Therefore, there is no transcriptional evidence that supports the existence of a unique mammary gland thiosterase that may contribute to the release of shorter fatty acids found in milk fat.

#### 3.3.2. Acyl-CoA Synthase Genes

**Figure 5:**
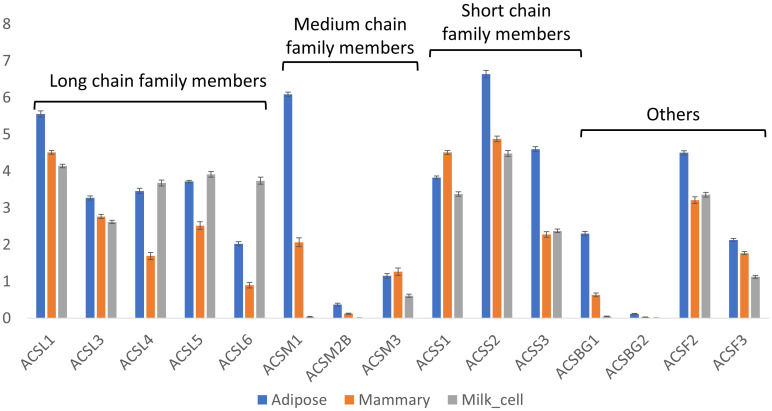
Expressions of acyl-CoA synthase genes. Expressions [Log(TPM+1)] of acyl-CoA synthetase genes in adipose and mammary tissues and milk cells. Means and error bars of all samples are shown by gene and tissue. Statistical analysis of the differences among the means of different tissues for each gene are provided in Supplementary File S3.

To utilize fatty acids, either for storage as neutral lipids or for other purposes (e.g., membrane lipids), ligation with a CoA group is needed. This step is catalyzed by acyl-CoA synthase genes, which have different isoforms with affinities for different chain lengths of fatty acids (e.g., ACSS, ACSM, and ACSL for acyl-CoA synthesis of short-, medium-, and long-chain fatty acids, respectively). In bovines, there are five isoforms of ACSL (ACSL1 and ACSL3-6), and their expressions are significantly different among tissues (Kruskal–Wallis test, *p* < 0.05, Figure 5). The expressions of all the isoforms were greater in adipose tissue than in mammary tissue (Steel–Dwass method, *p* < 0.05). In milk cells, three of the ACSL isoforms (ACSL4, 5, and 6)), had the highest expression levels (Steel–Dwass method, *p* < 0.05). However, it is not known if the unique fatty acid profile was maintained in these cells. Therefore, the implication of this unique expression pattern is not clear.

There are three isoforms of ACSM in bovine (ACSM1, ACSM2B, and ACSM3). ACSM2B and ACSM3 had low expressions in all tissues, while ACSM1 had the highest expression in adipose, which was four orders of magnitude higher than in mammary tissue.

Lastly, the expression levels of the ACSS genes with higher affinity for short-chain fatty acids (namely, ACSS1-3) were significantly greater in the adipose tissue (Steel–Dwass method, *p* < 0.05), except for ACSS1, which had the highest expression in mammary tissue (Steel–Dwass method, *p* < 0.05), with an expression level approximately 10-fold greater than that in adipose tissue.

#### 3.3.3. Triglyceride Synthesis Genes

**Figure 6:**
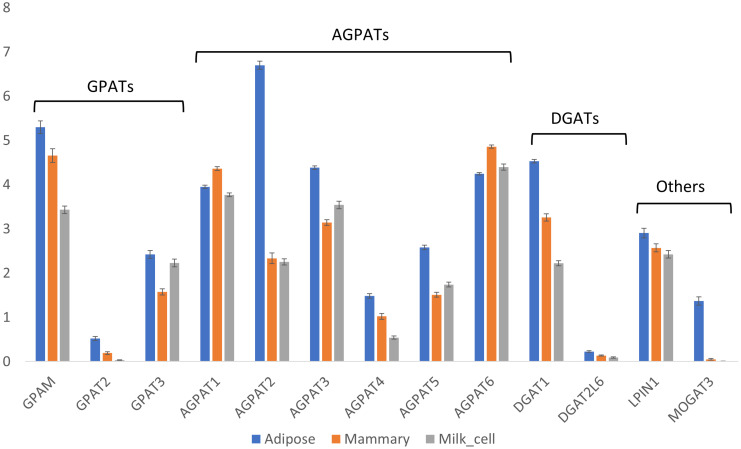
Expressions of TAG synthesis genes. Expressions [Log(TPM+1)] of triglyceride biosynthesis genes in adipose and mammary tissues and milk cells. Means and error bars of all samples are shown by gene and tissue. Statistical analysis of the differences among the means of different tissues for each gene are provided in Supplementary File S3.

Next, the expression of TAG synthesis genes was examined. These genes are involved in the esterification of fatty acid-CoA to a specific position on the glycerol backbone of TAGs. Thus, they may play a role in determining the composition of fatty acids simply by preferring certain types of fatty acids as substrates over others. Alternatively, rapid depletion of the cytoplasmic fatty acid-CoA pool due to its utilization for neutral lipid synthesis may shift the metabolic flux toward early chain termination of fatty acids. Therefore, differences in the expression patterns of the abovementioned genes can result in differences in TAG composition and fatty acid positional distribution among adipose and milk fat. Each step in the formation of TAGs is catalyzed by different families of genes, each with several members (isoforms). Considerable differences are found in the gene expressions of these members in different tissues (Figure 6). The first step in TAG formation is the esterification of a fatty acid to the sn-1 position of the glycerol backbone, which is catalyzed by GPAT (GPAM/GPAT1, GPAT2, and GPAT3). GPAM was the most abundant isoform in all analyzed tissues, while GPAT2 was almost undetectable. GPAM and GPAT3 had significantly higher expressions in adipose compared to mammary tissue (Steel–Dwaas method, *p* < 0.05; Figure 6).

The next step in the TAG synthesis pathway involves the esterification of a fatty acid to the sn-2 position of the glycerol backbone by AGPAT (AGPAT1-5, AGPAT6/GPAT4). This group of genes differed considerably among the tissues, and each tissue had a different blend of isoforms with differing ratios (Figure 6). In adipose tissue, AGPAT2 was the most dominant isoform, with significantly greater expression compared with all other tissues (Steel–Dwaas method, *p* < 0.05; Figure 6). In mammary tissue, the dominant isoform was AGPAT6, followed by AGPAT1, and both had the highest expression levels in mammary tissue, significantly differing from those in all other tissues (Steel–Dwaas method, *p* < 0.05; Figure 6).

The last step in TAG formation is the esterification of a third fatty acid to the glycerol backbone at the sn-3 position, which is catalyzed by DGAT. Interestingly, in bovine milk fat, short-chain fatty acids are almost exclusively esterified to this position [7]. Generally, two genes perform this step (DGAT1 and DGAT2); however, in bovines, only DGAT1 is currently annotated [21]. There is also another DGAT-like gene termed DGAT2L6, which has extremely low overall expression (Figure 6). Therefore, DGAT1 seems to be the dominant enzyme performing this final TAG synthesis step in all tissues. The expression of DGAT1 significantly differed between all tissue pairs (Steel–Dwaas method, *p* < 0.05), with the highest expression in adipose tissue, followed by mammary tissue, and milk cells (Figure 6). Taken together, the significant differences in the expression patterns of TAG synthesis genes may play a crucial role in the formation of unique dairy lipids.

## 4. Discussion

Unlike the more direct gene-to-protein relationship, the biosynthesis of lipids is the result of metabolic cycles that are more complex. Single-gene alterations in several organisms have managed to shift fatty acid synthesis toward the production of shorter-chain fatty acids [22,23,24]. Nevertheless, these alterations did not result in an oil composition that was sufficiently comparable with dairy fat; hence, it did not mimic its technological properties. To date, the mechanism by which short- and medium-chain fatty acids are produced in the bovine mammary gland is largely unknown. Therefore, this study aimed to narrow this knowledge gap by both enhancing our understanding of this unique biochemical process occurring in the mammary glands of ruminants and laying the foundation for translatable research that can contribute to the production of healthy, nourishing, and sustainable dairy alternatives.

As a first step toward deciphering the unique mechanisms underlying the composition of dairy fat, we applied a comparative approach and analyzed the gene expressions of three bovine lipogenic tissues. Our results show a clear separation of the liver samples from all other tissues in the PCA plot. This reflects the known physiological metabolic activity of the liver of ruminants, where TAG synthesis and beta-oxidation are more prominent than *de novo* fatty acid synthesis, which mainly occurs in adipose and mammary tissues [20]. We also noticed that the mammary samples were diverse along the PC2 scale, with some samples more similar to milk cells and others more similar to adipose tissue. A previous study showed that the majority of the somatic cells in milk are immune cells, and the minority are epithelial cells [25]. In addition, it was shown that some of the epithelial cells express markers of progenitor cells [26]. Therefore, these populations of milk cells are diverse, and while some cells can express a comparable transcriptome to that of lactating cells, others differ significantly from mammary tissue collected during lactation.

FASN plays a central role in fatty acid *de novo* synthesis and is responsible for short- and medium-chain fatty acid synthesis in the bovine mammary gland [7]. Observing correlation patterns rather than absolute gene expression can minimize the confounding effects of analyzing data from several experiments under different conditions, as was the case in this study. Therefore, we used FASN expression as a reference for fatty acid *de novo* synthesis and examined all other 734 lipogenic gene correlation trends with FASN.

Interestingly, in the mammary gland, approximately 70% of lipid metabolism-related genes were negatively correlated with FASN expression, and only 6.5% were positively correlated. Meanwhile, in adipose tissue, 18% and 22% of genes were negatively and positively correlated with FASN expression, respectively. Although it is difficult to determine the physiological meaning of such differences, it seems that the flow of metabolites in the lipogenic pathway has distinct kinetics in both tissues, which may contribute to the distinguished fatty acid composition of milk fat. The majority of lipogenic genes, including FASN, are under the same regulatory signals [27]. Therefore, the fact that the majority of these genes in the mammary gland were negatively correlated with FASN, a central enzyme in the fatty acid synthesis pathway, was unexpected. However, in accordance with this finding, it was shown previously that lipogenic genes in mouse mammary glands during lactation were not co-expressed [28]. Moreover, in the transition between pregnancy and lactation, the majority of genes are down-regulated in mouse mammary glands [29]. This may also occur in relation to lipid synthesis pathways; however, further research is warranted to verify this point.

Interestingly, in the mammary gland, DGAT1 and FASN were negatively correlated, which was unexpected because DGAT1 is a central gene in TAGs synthesis [30,31]. In a previous study on cows, a delayed peak in FASN expression on day 60 postpartum compared with DGAT expression, which peaks on day 15, was demonstrated [32]. The different temporal expression patterns of the two genes postpartum can be explained by the change in the main source of fatty acids for TAG synthesis throughout lactation. Throughout the lactation cycle, there is a shift from high long-chain fatty acid concentrations in the form of non-esterified fatty acids (NEFAs) toward high acetate availability in the serum [33]. In addition, diets rich in structural carbohydrates (i.e., forages) will increase the rumen fermentation production of acetate and butyrate, which are usually utilized by the mammary gland for the *de novo* synthesis of fatty acids and can shift the milk fat composition toward higher contents of short- and medium-chain fatty acids [34]. The increase in acetate requires a proper enzymatic response to efficiently utilize these building blocks for TAG synthesis and may explain why, according to our results, FASN and DGAT1 genes were not co-expressed in the mammary gland. At early lactation stages, there is a need to cope with large amounts of NEFAs derived from adipose lipolysis [33,35]. By rapidly utilizing NEFAs from the blood system for TAG synthesis, DGAT1 activity could protect cells from lipotoxicity [36]. Later, during the lactation cycle, with the elevated food consumption of cows, the acetate flux is elevated, along with reduced lipolysis and NEFA levels in the blood, which induces the *de novo* synthesis of fatty acids by FASN [35]. However, it remains unclear whether the negative correlation between FASN and DGAT1 is a result of lactation dynamics and temporal differences in the peak expression or whether it has a further role in the production of unique milk lipids, for example, the very short-chain fatty acids (C4:0 and C6:0), which are almost exclusively esterified to the sn-3 position [3] in a reaction catalyzed by DGAT1.

From all the genes that were related to lipid metabolism according to the GO annotation, we focused on genes from three categories that can directly contribute to the length of the fatty acids on TAGs. These categories consisted of the following: (i) Thioesterases were selected due to their direct involvement in the chain termination of fatty acids in other systems (i.e., plants [37] and rat mammary glands [38]). (ii) Acyl-CoA synthase genes are the link between the release of free fatty acids by FASN or their absorption from plasma and their incorporation into polar and neutral lipids. These enzymes have several isoforms with varying affinities to different chain lengths of fatty acids [39], giving them the potential to directly impact their downstream utilization. (iii) Triglyceride synthesis genes are directly linked to the composition and positional distribution of fatty acids on triglycerides [40], which are the main (~98%) component of milk lipids [1].

In many organisms, thioesterase activity is the main determinant of the chain length of *de novo* synthesized fatty acids. The chain termination of fatty acids typically requires the breaking of the thiol bond between the acyl chain and the ACP unit of FASN. In type I FASN (vertebrates), in most cases, this step is catalyzed by the thioesterase catalytic site of FASN. In some cases, such as in rats [38] or in the preen glands of aquatic birds [41], a separately encoded acyl-ACP thioesterase gene breaks the chain at an earlier phase, releasing shorter-chained fatty acids. Moreover, in plants and bacteria, which possess the separately encoded type II FASN, several acyl-ACP thioesterase genes may be involved in chain termination at various lengths [22,42,43]. However, in our mega-analysis, there was no transcriptional evidence to support the existence of a unique mammary gland thiosterase that may contribute to the release of shorter fatty acids found in milk fat. This finding supports the findings of Hansen and Knudsen (1980), which indicated that the *de novo* synthesis of short-chain fatty acids in the mammary gland is not dependent on thioesterase activity. In addition, this finding supports our hypothesis that the expression patterns of several genes in the lipogenic pathway contribute to the production of short-chain fatty acids.

Prior to utilization, all lipid intermediates must be activated by ligation to CoA. The genes responsible for the activation of hydrocarbon chains are divided according to their affinity to the carbon atom chain length and consist of short-, medium-, and long-acyl-chain family members. The ACSS1 gene is responsible for the conversion of acetate to acetyl-CoA [44] and thus might contribute to increasing the availability of acetyl-CoA in mammary epithelial cells, which can be utilized as a building block for *de novo* fatty acid synthesis by FASN. Evidence from fungi, plants [42], and animals, including dairy cows [11], implies that increasing acetyl-CoA availability compared with that of malonyl-CoA can increase the production of short-chain fatty acids. In our results, the expression of ACSS1 was the highest in the mammary gland, which may contribute to the availability of acetyl-CoA in the cytoplasm, and hence to the production of short-chain fatty acids. In addition, the expression of ACSL genes in the mammary gland were considerably lower than that in adipose tissue, and most of the isoforms were negatively correlated with FASN. Therefore, it seems that mammary gland metabolism favors the activation of acetate over long-chain fatty acids, which can contribute to increased levels of short-chain fatty acids.

Interestingly, it was demonstrated that the expression levels of ACSS1, which activates acetate, were elevated throughout lactation and peaked at day 120 postpartum [32]. This finding supports the increased utilization of acetate derived from rumen fermentation, which increases throughout lactation with elevated feed consumption compared with earlier stages of lactation [45]. Accordingly, fatty acid composition is altered throughout lactation, with elevated short- and medium-chain fatty acids at the advanced compared with the early stages of lactation [46]. These elevated acetate levels can be utilized for acetyl-CoA and short-chain fatty acid synthesis in the mammary gland.

Out of the 734 genes tested, only 29 genes were positively correlated with FASN uniquely in the mammary gland. Two genes stand out from this list of genes since they belong to the TAG synthesis category—AGPAT1 and AGPAT6. AGPAT1 was positively correlated with FASN in the mammary gland and negatively correlated with FASN in adipose tissue. Additionally, AGPAT6 had a strong positive correlation in mammary tissue, while in adipose tissue, no significant correlation was found between the two genes.

AGPATs are responsible for the utilization of the activated acyl-chain (i.e., acyl-CoA) and catalyzes its esterification to the sn-2 position of the glycerol backbone. In bovines, there are six isoforms of AGPAT that catalyze this step. Our results show that these genes were differentially expressed in the different tissues, with AGPAT2 being the dominant form in adipose tissue and the abovementioned AGPAT1 and AGPAT6 being the dominant forms in mammary tissue, as was also found in other studies [47].

The redundancy of these genes, together with their distinct expression patterns, may indicate that each gene evolved a slightly different function. These differences might include differences in substrate affinity, stereospecific regulation of the enzyme, or other factors. Together, they may be related to the different fatty acid compositions of different tissues. For example, for AGPATs that were isolated from lactating cow mammary glands, the affinity to butyric and caproic acid was extremely low; indeed, these fatty acids are rarely found at the sn-2 position [48]. On the other hand, DGAT1 from the bovine mammary gland was shown to prefer short-chain fatty acids as a substrate [49], which is in agreement with the fact that the majority of short-chain fatty acids found in milk are esterified to the sn-3 position. However, it should be noted that DGAT1 is expressed at relatively high levels in adipose tissue; therefore, the abundance of short- and medium-chain fatty acids in milk is probably not attributed to DGAT1 expression or activity but rather to the availability of these fatty acids in the mammary gland.

## 5. Conclusions

The unique composition of milk fats in dairy animals is responsible for many of the unique properties of dairy food products. However, attempts to mimic this process by engineering specific genes have not been successful, probably due to the complex multiple biosynthesis pathways involved in the production of fats with a unique fatty acid composition and stereospecific distribution. The findings of the present study suggest that independent of the unique bovine FASN properties, two other factors support the production of unique fats: (i) an enzymatic infrastructure that prioritizes the ligation of CoA to acetate for the production of short-chain fatty acids over the ligation of CoA to long-chain fatty acids for their utilization; and (ii) a unique expression pattern of TAG synthesis genes, which likely impacts the downstream utilization and positional distribution of milk TAGs. Therefore, the unique fatty acid composition and positional distribution of bovine milk fats are a result of the combination of the abovementioned properties of the mammary gland.

## Figures and Tables

**Table 1:** Types of genes with positive correlation with FASN gene expression in adipose and mammary tissues.

Category	Mammary	Shared	Adipose
Availability of building blocks for fatty acid *de novo* synthesis		2	18
Acyl-CoA thioesterases			3
Elongase/desaturase		1	6
Acyl-CoA synthase		2	5
Phospholipid/sphingolipid biosynthesis	2	1	2
TAG synthesis	2	1	5
Others	25	11	104

## Data Availability

The original contributions presented in the study are included in the article or Supplementary Materials. Further inquiries can be directed to the corresponding authors.

## Supplementary Materials

The following supporting information can be downloaded at https://www.mdpi.com/article/10.3390/foods14030412/s1: Supplementary File S1. The final dataset used for this study, including the genes, gene accession numbers, and their assigned category (as described in the Methods section). Additional information of samples and sample meta-data is provided, along with expression data in the form of raw counts, TPM, and Log(TPM+1). Supplementary File S2. Summary statistics for all genes by tissues (adipose, mammary, and milk cells), including Kruskal–Wallis test results for differences among tissues for each gene. Supplementary File S3. Steel–Dwass method comparisons between all tissue pairs for all genes. Supplementary File S4. Correlation coefficients (r) and their *p*-values between FASN and selected lipogenic genes in adipose and mammary tissues.
